# *Xylella fastidiosa* in Olive: A Review of Control Attempts and Current Management

**DOI:** 10.3390/microorganisms9081771

**Published:** 2021-08-19

**Authors:** Massimiliano Morelli, José Manuel García-Madero, Ángeles Jos, Pasquale Saldarelli, Crescenza Dongiovanni, Magdalena Kovacova, Maria Saponari, Alberto Baños Arjona, Evelyn Hackl, Stephen Webb, Stéphane Compant

**Affiliations:** 1 Consiglio Nazionale delle Ricerche, Istituto per la Protezione Sostenibile delle Piante, Sede Secondaria di Bari, 70124 Bari, Italy; massimiliano.morelli@ipsp.cnr.it (M.M.); pasquale.saldarelli@ipsp.cnr.it (P.S.); maria.saponari@ipsp.cnr.it (M.S.); 2 DMC Research Center, 18620 Granada, Spain; jgmadero@dmcrc.com (J.M.G.-M.); abarjona@dmcrc.com (A.B.A.); 3 Área de Toxicología, Facultad de Farmacia, Universidad de Sevilla, 41012 Seville, Spain; angelesjos@us.es; 4 Centro di Ricerca, Sperimentazione e Formazione in Agricoltura Basile Caramia, 70010 Locorotondo, Italy; enzadongiovanni@crsfa.it; 5 RTDS Group, 1080 Wien, Austria; maggiekovacova@gmail.com (M.K.); webb@rtds-group.com (S.W.); 6 AIT Austrian Institute of Technology, Center for Health and Bioresources, 3430 Tulln, Austria; evelyn.hackl@ait.ac.at

**Keywords:** bacterial disease, olive outbreak, sustainable control, IPM strategy, *Philaenus spumarius*

## Abstract

Since 2013, *Xylella fastidiosa* Wells et al. has been reported to infect several hosts and to be present in different areas of Europe. The main damage has been inflicted on the olive orchards of southern Apulia (Italy), where a severe disease associated with *X. fastidiosa* subspecies *pauca* strain De Donno has led to the death of millions of trees. This dramatic and continuously evolving situation has led to European and national (Italian and Spanish) measures being implemented to reduce the spread of the pathogen and the associated olive quick decline syndrome (OQDS). Research has been also carried out to find solutions to better and directly fight the bacterium and its main insect vector, *Philaenus spumarius* L. In the course of this frantic effort, several treatments based on chemical or biological substances have been tested, in addition to plant breeding techniques and integrated pest management approaches. This review aims to summarize the attempts made so far and describe the prospects for better management of this serious threat, which poses alarming questions for the future of olive cultivation in the Mediterranean basin and beyond.

## 1. Introduction

*Xylella fastidiosa* Wells et al. (*Xanthomonadaceae*) [1] is a bacterial pathogen that has been well documented for its worldwide spread and infection of a broad range of plant species. The global distribution of this pathogen continues to increase due to anthropogenic movements of goods and plant materials. Recent surveys have shown several incursions (ingresses) of this pathogen in Europe and the Mediterranean basin [2].

Different subspecies of *X. fastidiosa* are known, which are described according to the host range and genetic relationships [3]. Indeed, the bacterium is highly polyphagous, infecting 638 plant species, and in several cases without causing symptoms [4]. Currently, three subspecies of *X. fastidiosa* (*fastidiosa*, *multiplex,* and *pauca*) are generally accepted by the scientific community to be the main grouping, although there is no type strain available for subspecies *pauca* in the public databases [5]. Proposals also exist for the establishment of the subspecies *sandyii*, *morus,* and *tashke* [6], and the situation is continuously evolving due to the fast acquisition of new genomic information and strain distinction by multilocus sequence typing [7].

Among the new *X. fastidiosa* plant pathosystems, infections in olive plants during recent years (Table 1) have had a very devastating outcome because of the severe symptoms of olive quick decline syndrome (OQDS), which was first described in 2013 [8]. In susceptible cultivars, *X. fastidiosa* infestations have led to extensive desiccation, which results in widespread tree die-off. Discovered in Salento, Apulia, Italy, *X. fastidiosa* subspecies *pauca* strain “De Donno” ST53 [9,10] has quickly spread from its original infection zone, due to the very efficient and highly prevalent vector *Philaenus spumarius* L., a xylem sap feeder insect (Hemiptera: Aphrophoridae) [11]. In Italy, the spread of *X. fastidiosa* has been greatly facilitated by favorable vector conditions, the planting of extensive monocultures of two autochthonous susceptible olive cultivars (Cellina di Nardò and Ogliarola salentina), and the current dense population of *X. fastidiosa* on infected olive trees [12].

Since its first detection, *X. fastidiosa* subspecies *pauca* strain “De Donno” has infected about 4 million trees in the outbreak area [13]. This has caused, and is still causing, enormous economic losses of olive trees and oil production outputs, in addition to dramatic changes in the Mediterranean landscape, with olive trees being a strongly ingrained part of the cultural heritage and an important element of the flourishing tourism industry. Two years after its discovery in Apulia, *X. fastidiosa* was considered no longer eradicable due to the large occurrence of infected plants in the outbreak area. This induced the National and European Phytosanitary Authorities to move from an “eradication” to a “containment” strategy [14]. Its recent detection in the mid-Apulian region close to the area of monumental olive trees in 2020 [15] caused great public alarm because of the high value of these millennial trees, which besides being protected by regional law, have been proposed to have UNESCO heritage status.

Genetic studies of the *X. fastidiosa* strain “De Donno” suggest that this pathogenic strain was introduced and not native to the Mediterranean region. Phylogenetic analysis indicates that the “De Donno” strain is closely related to a strain of *X. fastidiosa* from Costa Rica, which is capable of infecting oleander (*Nerium oleander* L.) and coffee (*Coffea* spp.) plants. Based on this information, it is suspected that the introduction of *X. fastidiosa* to the Mediterranean region resulted from the importation of ornamental plants [16,17]. Moreover, after performing an extensive study of the genomic diversity of several *X. fastidiosa* subspecies, the introduction of the olive strain ST53 in Apulia was dated back to 2008 [18].

*Xylella fastidiosa* was first reported in Spain in 2016 in the Balearic Islands. Three subspecies (*multiplex*, *fastidiosa,* and *pauca*) and 4 sequence types (*multiplex* ST81 and ST7, *fastidiosa* ST1 and *pauca* ST 80) have infected more than 20 plant species, including grape (*Vitis* spp.), almond (*Prunus* spp.), olive (*Olea europaea* L.), and fig (*Ficus* spp.), on all islands except Formentera [19]. According to a recent study, 79.5% of the almond trees on the islands (approx. 1,250,000 trees) have been infected by *X. fastidiosa* [20]. In the Valencian Community, *X. fastidiosa* was first noticed in 2017 on almond trees (unidentified species). In December 2020, more than 600 wild or cultivated olive trees were reported as being positive for *X. fastidiosa* in the Balearic archipelago [19]. According to the Generalitat Valenciana (the government of Valencian Autonomous Community), 18 plant species have been infected so far, although 93.6% of the samples found infected were from almond trees (*Prunus dulcis* (Mill.) D. A. Webb). In 2021, the total area declared infected across the whole community reached 2292 ha., with more than 100,000 almond trees having been destroyed [21]. In 2018, a single olive tree grown in the Madrid area was tested positive for *X. fastidiosa* subsp. *multiplex* (ST6) [22]. In the same year, *X. fastidiosa* was detected in three specimens of *Polygala myrtifolia* L. in a nursery of ornamental plants in Almeria (Andalusia), although currently it is considered to be already eradicated at this site [20].

In late 2018, a new outbreak of *X. fastidiosa* in Italy was reported in Tuscany [23]. Multilocus sequence typing and genomic analyses of isolates infecting different ornamental plants and almond trees revealed the occurrence of the newly discovered ST87, belonging to subsp. *multiplex* and phylogenetically related to all the variants detected in the EU [24]. In France, *X. fastidiosa* subsp. *multiplex* has been reported in Corsica (2015), Provence (2019), and Occitanie (2020). Lavender (*Lavandula* spp.), ornamental plants, myrtle shrub (*Myrtus communis* L.), rosemary (*Salvia rosmarinus* Spenn.), and broom (*Spartium junceum* L.), as well as two olive trees, were found to be infected by *X. fastidiosa* subsp. *pauca*, although this was promptly eradicated. The outbreaks in the French regions seem to be due to several introductions during the 1980s [25].

A more recent introduction occurred in 2019 in Portugal, where *X. fastidiosa* subsp. *multiplex* ST7 was found in some asymptomatic plants of French lavender (*Lavandula dentata* L.). Although more extensive surveys have led to the identification of about 50 other host species within the country, so far only four olive trees have been found to be infected and promptly uprooted [4].

As in other *X. fastidiosa* epidemics, such as Pierce’s disease (PD) of grapevine and citrus variegated chlorosis, the current disease management approach relies on control activities that integrate agronomical (soil tillage and weed elimination) and chemical interventions. Measures aimed at reducing the vector populations involve the elimination of the sources of inoculum, which in many places in Apulia are centenary olive trees protected by law [26]; however, such measures are currently not sufficient. The search for a cure for *X. fastidiosa*-infected plants is an ongoing process to which not only academics, professionals, and growers but also ordinary citizens continuously contribute (for example by searching for spontaneous olive seedlings surviving from *X. fastidiosa* infection in the epidemic area). Due to the severity of induced disease and the economic importance of the susceptible crops, the discovery of an efficient solution would be self-promoting among people who make a living from agriculture.

The present review lists most of the attempts made so far to combat *X. fastidiosa* in olive plants, distinguishing between different types of control strategies used, including containment or eradication measures taken by the EU and national governments, direct *X. fastidiosa* and vector control, plant breeding, and integrated pest management approaches. In addition, we report on *X. fastidiosa* infection in almond plants and the containment measures implemented in Spain, because in this area, as in Italy, the bacterium is severely affecting a major crop. The limited outbreaks identified in France and Portugal, which have mainly occurred on ornamental plants, are not considered as they are outside the scope of the present review. Prospects for better control are further discussed, as the threat posed by *X. fastidiosa* to European agriculture, and particularly to olive, demands urgent solutions. Additionally, the proposed solutions are bound to comply with the sustainability principles expressed by the European Commission (EC) in the European Green Deal.

## 2. Containment and Eradication Measures

### 2.1. Xylella fastidiosa and EU Legislation

In response to the appearance of the *X. fastidiosa* outbreak in Italy, the EC took necessary action as soon as possible to prevent the introduction and spread of *X. fastidiosa* by enforcing the Commission Implementing Decision (EU) 2015/789 [14]. This legislative act detailed contingency plans, the establishment of demarcated areas, containment measures, eradication measures, and last but not the least, protection measures against *X. fastidiosa*. The following year, in 2016, the EC decided to continue regulating *X. fastidiosa* within the European Union as a quarantine pest under Regulation (EU) 2016/2031 [27], in continuity with the previous Directive 2000/29 [28].

**Figure 1:**
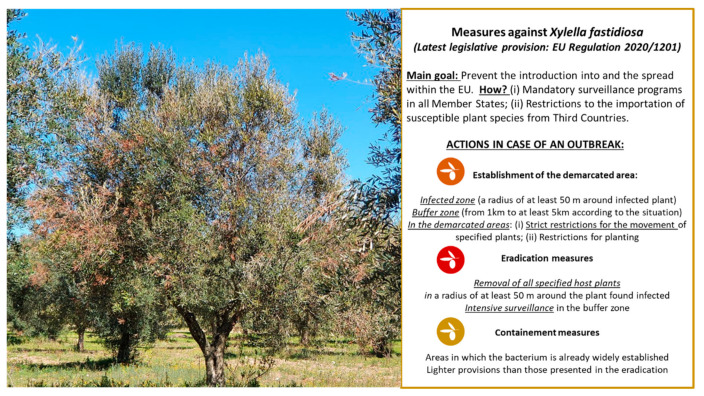
Measures of containment/eradication against *Xylella fastidiosa*.

With the continuous spread of *X. fastidiosa* in Europe, on the 14th of August 2020, the EC decided to repeal (EU) 2015/789 [14] by introducing the new Regulation (EU) 2020/1201 [29], which included new and adapted control measures based on the latest findings from the European Food Safety Authority (EFSA). In summary, Regulation (EU) 2020/1201 [29] mandates that upon the detection of an outbreak due to *X. fastidiosa* in any EU member state, the relevant plant health authorities within that member state shall, without delay, establish a demarcated area, which includes an infected zone and a buffer zone. According to article 4 (EU) 2020/1201 [29], the infected zone shall have a radius of at least 50 m around the *X. fastidiosa*-infected plants. The buffer zone shall have a width of at least 5 km in the case when an infected zone established is subject to containment measures, as set out in articles 12 to 17; a width of at least 2.5 km in the case when an infected zone is subject to eradication measures, as set out in articles 7 to 11; and a width of 1 km for isolated *X. fastidiosa* outbreaks where eradication measures have been immediately taken (Figure 1).

The established width of 5 km for the infected zones within the demarcated areas subject to containment measures has sparked discussions amongst scientists and farmers, as this is an adapted measure from the previous Commission Implementing Decision (EU) 2015/789 [14]. It has caused concerns over its efficacy, as the 5 km buffer zone might not be sufficient in containing the spread of *X. fastidiosa*, in comparison to the previously mandated widths of 20 km (containment zone) and 10 km (buffer zone), as per (EU) 2015/789 [14].

Regarding eradication measures within articles 7 to 11 of (EU) 2020/1201 [29], all infected or symptomatic plants have to be removed, including those that belong to the same species as the infected plants (irrespective of their current plant health status), as well as other plants susceptible to *X. fastidiosa* within the infected zone. All other host plants within the 50 m infected zone must be sampled and tested for *X. fastidiosa*. Regarding the 2.5 km buffer zone, the member state’s relevant authority shall further test and sample the host plants, including intensive surveillance. The surveys shall take into consideration the EFSA Guidelines on Risk-Based and Statistically Sound Surveys [30].

Regulation (EU) 2020/1201 [29] also details specific containment measures under articles 12 to 17 for regions where eradication of *X. fastidiosa* is not feasible. These include Southern Apulia (Italy), Corsica (France), and the Balearic Islands (Spain). Within these regions (and within the infected zones), all infected plants shall be removed followed by intensive surveillance within an area measuring at least 5 km from the border of the infected zone with the buffer zone. For the 5 km buffer zones, the eradication measures apply (Figure 1).

Lastly, Regulation (EU) 2020/1201 [29] also includes measures related to the planting of specified plants within the infected zone, the movement of plants within and out of the demarcated areas, as well as control measures to prevent the introduction of *X. fastidiosa* into the European Union.

With more findings being shared by EFSA and other EU research projects related to *X. fastidiosa*, further adaptations of EU provisions regarding the spread of *X. fastidiosa* in the EU territory can be expected in the near future.

### 2.2. Italy

Regarding EU regulations, and since the first detection of *X. fastidiosa* in Apulia in 2013, several legislative decrees have been issued by the Italian Ministry of Agriculture, Food, and Forestry to detail the emergency measures proposed to prevent, control, and eradicate *X. fastidiosa* from the Italian territory, in compliance with EU Regulation 2015/789 [14] and successive modifications. The application of measures is assigned to the Regional Phytosanitary Services operating in synergy with and referring to the Central Phytosanitary Service. The basic principles translated in the Italian legislation are currently: (1) the distinction of “host” (species susceptible to all *X. fastidiosa* subspecies worldwide) and “specified” (species susceptible to the local *X. fastidiosa* genotype) plants; (2) control measures in Apulia, which were soon changed from “eradication” to “containment”; (3) territory demarcation in infected and buffer zones as detailed above. Based on these principles, the emergency plan currently consists of monitoring plant infection status in the buffer zone and monitoring the insect vector (*P. spumarius*) life stage to plan agronomic and chemical control measures. Moreover, the planting of susceptible species in the infected area is prohibited, except for species or cultivars found to be resistant or tolerant, along with the prohibition of movement of “host” and “specified” plants out of the demarcated area. The results of monitoring, legislative documents, scientific advances, and other information are regularly updated on the freely accessible Apulia regional website dedicated to the *X. fastidiosa* emergency [31].

### 2.3. Spain

In Spain, the MAPA (Ministerio de Agricultura, Pesca y Alimentación) Contingency Plan for *X. fastidiosa* was elaborated for the first time in 2015, which was revised for the last time in October 2020 by the Ministry of Agriculture, Fisheries, and Food after the publication of EU Regulation 2020/2021 [32]. The plan provides specific guidelines regarding: (1) the organization and responsibilities of the interested groups involved in the plan; (2) legal provisions for the pest, background, and symptoms; (3) relevant factors regarding the prevention, detection, damage, and control of the pest; (4) containment and eradication procedures, including official measures. Legal provisions to consider include EU regulations, national laws, and autonomic regulations such as the ones established in the Balearic Islands, Valencian Community, Community of Madrid, and Andalusia. Competences in this plan are distributed among the MAPA, autonomous communities (forest and plant health official bodies), their diagnostic laboratories, and also the national reference laboratories.

Upon detection of an outbreak, the competent bodies of the autonomous communities should establish an Emergency Management Team to deal with tactical and operational aspects of the contingency plan or specific action plans. These plans are executed when the pathogen/disease is detected as a result of a general inspection, specific surveys, when competent authorities are informed of its presence by an operator or individual, or due to import or movement of plants.

When an autonomous community suspects the presence of an outbreak of *X. fastidiosa*, a series of precautionary measures included in the contingency plan must be further adopted [32]. Once confirmed by the official laboratory or a national reference laboratory, the detection of the outbreak should be reported to the MAPA and measures included in the contingency plan to prevent the spread of the pathogen and to achieve its eradication should be adopted. Moreover, the European Commission and the other member states must also be informed. Demarcated areas are then established, delimiting an infected zone and a buffer zone following the guidelines established in Regulation (EU) 2020/1201 [29] and where eradication measures must be adopted. Additionally, the competent official bodies (MAPA and the affected Autonomous Community) must establish a plan that provides information on the disease and the contingency plan must be published on the websites of these bodies. This information must be widely distributed to all stakeholders, the general public, travelers, professionals, and international transport companies.

### 2.4. Considerations for Existing Measures in the Framework of the “Farm to Fork Strategy”

Following the recent EU and national provisions regarding *X. fastidiosa*, which rely on the control of the vector and inoculum sources, the development of biopesticides to target the bacterium and its vector is of paramount importance to help reduce the spread of the pathogen in Europe. Most noteworthy is the reduced width of the infected zone in areas where containment measures apply, which may facilitate the spread of the disease. In light of such fears, the relevant authorities of the Apulia region have decided to maintain the old demarcation areas as mandated by the initial Commission Implementing Decision (EU) 2015/789 [14]. More studies and observations are needed to determine the efficacy of the abovementioned decisions. As such, should the worst-case scenario become reality and the newly adopted (EU) 2020/1201 [29] 5 km width of the infected zone prove not to be efficient in preventing the spread of *X. fastidiosa*, the introduction of curative and preventive solutions for *X. fastidiosa*-infected plants might be the only effective solution regarding the containment and eradication of *X. fastidiosa*.

Once novel biopesticides are introduced and production is upscaled to the market, the EU legislation on protective measures against *X. fastidiosa* might be more relaxed in the near future.

Within the framework of the recently published Farm to Fork Strategy [33], the heart of the European Green Deal, the EC is determined to reduce the use and risk of chemical pesticides; thus, there is an urgent need to introduce low risk and non-chemical pesticides as alternative means to deal with dangerous pathogens.

## 3. Current Attempts to Control *X. fastidiosa* in Olive—State of the Art

Since the first identification of *X. fastidiosa* strain “De Donno” in olive trees [34] and the observation of the devastating damage caused in Apulian orchards by the associated disease [10], researchers have produced a growing body of literature on the attempts to control the pathogen, in addition to government measures, through the application of different treatments [35]. An extensive literature search of the approaches that have been taken within a fairly limited time returned a satisfactory number of experimental attempts, including in vitro studies and the application of potential treatment solutions directly to affected olive trees. Most experiments to control *X. fastidiosa* on olive trees have been performed in Italy.

**Figure 2:**
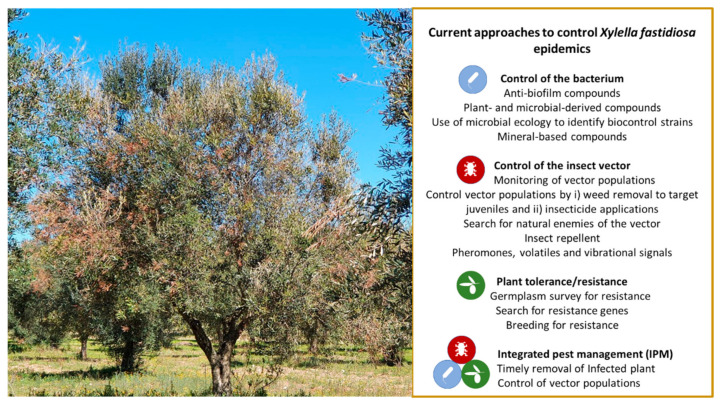
Current approaches to control *Xylella fastidiosa* epidemics.

The different studies have relied on several control approaches, which in turn involve mineral formulations, chemical compounds, natural products, and microbial antagonists (Figure 2, top). It is worth noting that given the recent introduction of this highly virulent strain of *X. fastidiosa* in Apulia, most of these studies are considered to be at the preliminary stage, although in the absence of an effective strategy, some of these attempts appear to be at least promising.

### 3.1. Minerals and Compounds Control: Moving beyond Conventional Agrochemicals

A series of studies conducted in vitro [36,37,38] showed that alterations in mineral homeostasis, mainly involving zinc, copper, and calcium ions, may have significant effects on *X. fastidiosa* Temecula1, which is responsible for PD in grapevine, affecting relevant biological features, such as the biofilm formation and growth rate, and possibly interfering with the expression of its virulence traits in the host tissues. Based on these studies, recent experiments aimed to investigate how the plant ionome, i.e., the relative content of mineral elements found in a specific tissue [39], could interfere with the expression of symptoms caused by the *X. fastidiosa* strain “De Donno” in olive trees. Interestingly, pursuing this approach, D’Attoma et al. [40,41] provided evidence that higher contents of calcium and manganese may contribute to resistance traits shown by the cultivar Leccino. Relying on a similar strategy, Del Coco et al. [42] investigated the perturbation of the ionomic profile of the leaves, following treatment of *X. fastidiosa*-infected trees with Dentamet^®^, a zinc–copper–citric acid biocomplex. This study corroborated the evidence gathered from a parallel approach that relied on a metabolomic analysis to reveal substantial changes in the metabolic profiles of *X. fastidiosa*-susceptible olive cultivars, following crown treatment with Dentamet^®^ complex [43]. It should be noted that the earliest descriptions of the application of Dentamet^®^ via foliar spray had shown that this complex can reduce *X. fastidiosa*-associated disease severity; however, the restricted time range of the application and the limited number of observations did not allow for conclusive evidence of complete eradication of the pathogen [44]. A further mid-term assessment revealed that the bacterial concentration tended to decrease in trees regularly sprayed with the biocomplex over 3–4 years [45].

Aside from the administration of zinc and copper, other strategies to control *X. fastidiosa* in olive plants and employing mineral solutions have been attempted in Italy. When sprayed with ammonium chloride, OQDS-affected trees showed clear symptom reductions; however, no significant differences in the bacterial populations were observed [46]. Recently, metal nanooxides have also been explored as carriers for the direct release of phytodrugs targeting *X. fastidiosa* in olive plants. Transmission electron microscopy observations showed an alteration of the bacterial cell wall following the interactions with calcium carbonate nanocarriers, which were absorbed by the olive roots and successfully translocated to conductive tissues [47].

Among the most well-studied control strategies for *X. fastidiosa* is also N-acetylcysteine (NAC). This mucolytic cysteine analogue, used mainly to treat human diseases [48], had shown promising inhibitory effects on *X. fastidiosa* strain 9a5c and its associated disease in sweet orange plants [49]. Building on this experience, some field trials were performed in Apulia to verify the NAC effect on OQDS. In general, treatment with NAC seems to decrease disease progression, especially using NAC endotherapy; however, qPCR assays did not show any significant reduction in the bacterial population size [50]. When investigating its effect on *X. fastidiosa* strain “De Donno” regarding in vitro behavior, Cattò et al. [51] found, however, that sub-lethal concentrations of NAC had a significant effect on *X. fastidiosa* biofilm formation, inducing a hyper-attaching phenotype, with potential impacts on strain virulence and vector acquisition.

Other approaches to reduce *X. fastidiosa* involved fosetyl–aluminum nanocrystals coated with chitosan [52] antimicrobial peptides (AMPs) [53], however, so far such mineral solutions have not led to efficient *X. fastidiosa* disease control and further products are still needed.

None of the mineral-based approaches have proven sufficient to control the bacterium *in planta*; therefore, none of the approaches have been validated for use in the current management strategy. Consequently, no data exist regarding the development of *X. fastidiosa* resistance to the applied minerals or on potential effects on the olive microbiota. As many of these minerals or compounds have significant in vitro effects on the bacterium lifestyle or survival, future research trends should consider optimizing their delivery to better target *X. fastidiosa* in the xylem network.

### 3.2. Plant- and Microbial-Derived Compounds

To explore the use of natural products from plants or microorganisms, Bleve et al. [54] evaluated the in vitro antimicrobial activities of different classes of plant-derived phenolics compounds (4-methylcathecol, cathecol, veratric acid, caffeic acid, and oleuropein), filtered fractions of olive mill wastewaters (OMW), *Trichoderma* spp. culture extracts, and fungal toxins. All tested phenolic compounds showed some inhibitory activities against *X. fastidiosa* strain “De Donno” isolated from olive plants, although limited to reversible bacteriostatic effects. Moreover, ophiobolin A and gliotoxin showed bacteriostatic inhibition, whereas a crude culture extract from a strain of *Trichoderma citrinoviridae* exhibited bactericidal properties. Interestingly, the addition of microfiltered OMW fractions in the growth medium impacted “De Donno” growth.

Several phenolic compounds, such as coumarins, stilbenes, and flavonoids, have been evaluated using in vitro assays for their potential use against PD-associated *X. fastidiosa* strains. Overall, these phenolic compounds were effective in inhibiting *X. fastidiosa* growth, as indicated by low minimum inhibitory concentrations. In addition, phenolic compounds with different structural features exhibited different antagonistic capacities. Particularly, catechol, caffeic acid, and resveratrol showed the highest inhibitory potential against the pathogen [55]. In similar assays, in vitro activity was evaluated in different concentrations of phenolic compounds, such as gallic acid, epicatechin, and resveratrol, on the growth of *X. fastidiosa*. Although none of these compounds inhibited bacterial growth in a significant way, some of them, such as epicatechin and gallic acid, reduced cell surface adhesion. In addition, cell–cell aggregation decreased with resveratrol treatment [56].

Plant oils and extracts of multiple botanical species have also been tested. They are among the constituents of NuovOlivo^®^, a natural bioactive detergent, which was also tested for its effectiveness in controlling OQDS. Spray treatments based on a small number of samples and a limited time span lowered the disease index and *X. fastidiosa* DNA levels, in addition to inducing plant defense and protecting cell membrane integrity [57], although more field assays should be carried out to sufficiently prove the potential of the product.

An attempt to reduce *X. fastidiosa* and its associated plant symptoms was made based on its diffusible signal factors. The lifecycle of *X. fastidiosa* proved to be finely regulated by a complex metabolic pathway regulated by a family of short-chain fatty acid molecules known as diffusible signal factors (DSF) [58,59], whose potential application for biological control of *X. fastidiosa*-associated diseases has been extensively investigated in grapevine and citrus plants [60,61]. New experiments are now underway pursuing the chemical characterization of DSF produced by the strain “De Donno”, in order to identify chemical analogues to be used for modulating cell-to-cell signaling and to help reduce the impacts of *X. fastidiosa* infections on olives [62].

### 3.3. Microbial Control of X. fastidiosa Infections

This plant hosts different microorganisms in its plant organs. Differences between susceptible and resistant/tolerant varieties for a disease have been correlated to the plant microbiome [63,64]. The unprecedented interest in microbial endophytes as biocontrol agents against pathogens [63,65], together with some promising, although preliminary, indications of their use in the control of strain Temecula1 [66,67,68], has boosted the quest for similar solutions to be applied against *X. fastidiosa* strain “De Donno” and to mitigate the effects of the associated disease.

Recently, two studies explored the potential role of microbial endophytes in contributing to the expression of resistance traits against OQDS, which was described with some olive cultivars. Vergine et al. [69] observed an overt dysbiosis established by *X. fastidiosa* infection in the susceptible cultivar Cellina di Nardò, which was not found in the resistant cultivar Leccino, whose microbial communities showed a greater diversity. The tendency of the endophytic microbiome to succumb to the occupation of the whole ecological niche by *X. fastidiosa* as the infection progresses was confirmed in the analysis by Giampetruzzi et al. [70], who found that this trend was more evident in the susceptible cultivar Kalamata than in the resistant FS-17^®^. Albeit differences in the microbiomes of the susceptible versus resistant cultivars were observed, when evaluating the biocontrol potency of several endophytic bacterial strains isolated from olive trees located in the *X. fastidiosa*-affected area, none of them proved to be efficient in inhibiting “De Donno” growth [71]. Similar efforts to identify potential biocontrol agents within the endophytic microbial communities inhabiting *X. fastidiosa*-infected olive trees led, however, to the discovery of *Methylobacterium mesophilicum* and *M. radiotolerans* strains found to be able to produce extracellular siderophores. Microorganisms producing these Fe^3+^-binding compounds can significantly enhance their biocontrol efficiency [72]; therefore, investigations are being conducted to evaluate *in planta* and in vitro growth competition assays and the effects of these naturally occurring *Methylobacterium* strains on “De Donno” populations [73].

Among other microbial strains, the beneficial endophyte *Paraburkholderia phytofirmans* PsJN, isolated from onion roots [74,75], is known to colonize several host plants [75,76], stimulating their growth and protecting them against biotic and abiotic stresses [76]. It has been shown to be effective in reducing Pierce’s disease symptom severity and *X. fastidiosa* Temecula1 populations in grapevines [77]. Preliminary trials aimed at testing its effectiveness as a biocontrol agent in the “De Donno” olive pathosystem in Italy, although limited to a single season, have not revealed significant differences in the reduction of OQDS symptoms in therapeutic treatments, nor reduction of the new infections upon preventive applications [78]. Other microbial strains are also currently being tested for control of *X. fastidiosa* under field conditions; however, so far no validated microbial-based solution is currently available for farmers, pinpointing that efforts need to also be directed toward other approaches to reduce the pathogen load in olive trees.

## 4. Current Attempts to Control the Insect Vector(s) in Olives—State of the Art

Vector control is the principal method available for controlling many insect-borne diseases [79]. The lack of therapeutic applications to cure plants infected by *X. fastidiosa* makes vector control the main option available to limit the spread of the pathogen in contaminated areas (Figure 2, middle). Given the unique mechanism (persistent non-circulative and without a latency period) underlying the insect transmission of *X. fastidiosa* and the difficulties in interfering or disrupting the acquisition and transmission processes, vector control aims to limit the transmission by reducing or eliminating the vector populations visiting susceptible host plants.

### 4.1. Survey of the Insect Vectors

Surveys for potential insect vectors of *X. fastidiosa* in Europe and the Mediterranean countries have unambiguously indicated spittlebugs (Hemiptera: Aphrophoridae), mainly those belonging to the genus *Philaenus*, as the dominant xylem sap feeders in olive groves [80]. Currently, the only confirmed vectors of *X. fastidiosa* in Europe are *P. spumarius* (Hemiptera: Aphrophoridae), and under experimental conditions, *Neophilaenus campestris* Fallén and *Philaenus italosignus* Drosopolous and Remane [26]. Before the emergence of *X. fastidiosa* in Europe, these insect species were neglected and poorly investigated, as they were never associated with significant direct damage to crops. Nowadays, because of their primary role as the European vectors of *X. fastidiosa*, they have gained major attention from the scientific community [81]. The outcomes of several studies are now available, disclosing relevant information on their biology and ecology (e.g., feeding behavior, host preference, bacterial transmission efficiency, population dynamics) and providing important hints to implement effective control strategies [81,82].

Spittlebugs are univoltine, overwintering as eggs and with a development cycle consisting of a pre-imaginal stage (five instars) before the emergence of the adults. Control strategies can target both nymphal and adult populations, although applications targeting the nymphs are more effective and sustainable. The nymphs have limited movement ability, and under the current epidemiological scenarios in the European outbreaks, do not contribute to the spread of the infections [81]. Adults can acquire and transmit the bacterium soon after feeding on infected plants (without a latency period) and throughout the whole life span (i.e., from May to October, depending on the climatic conditions), thereby challenging the effectiveness of chemical control to protect plants from the infections during the whole adult season.

Bodino et al. [80] described the stage-structured populations of nymphs and adults in Apulian olive groves, allowing determination of the best time to apply control measures. According to their observations, the newly hatched nymphs (1st instar) always disappeared just before the peak of the 4th instar nymphs, while the first adults were only captured after this peak; therefore, any control measure applied at the 4th instar peak could potentially target the whole nymph population before the onset of the adults, achieving the maximum efficacy.

### 4.2. Weed Management

With regard to the specific interventions used to reduce the juvenile populations, several trials have been conducted in Italy and Spain under laboratory, semi-field, and field conditions. More specifically, Dongiovanni et al. [83] compared over three years the effectiveness of different applications targeting weeds and ground vegetation as means to reduce juveniles of *P. spumarius* and *N. campestris*. The following trials were compared: (i) no tillage; (ii) soil tillage performed twice (in early winter and in spring when the majority of the nymphs were at the IV instar); (iii) soil tillage performed only during winter; (iv) sowing *Poaceae* crops (*Lolium* spp. L. and *Hordeum vulgare* L.); (v) shallow ploughing; (vi) mulching; (vii) application of herbicides; (viii) pyroweeding. The population densities recorded (nymphs/m^2^) in these trials clearly showed that regardless of the interventions, all were able to significantly reduce the juveniles of both spittlebug species compared to the non-disturbed ground vegetation (no tillage). More specifically, the use of a systemic herbicide, pyroweeding, and double tillage yielded the highest efficacies for both spittlebug species. Tillage performed in winter was effective for *N. campestris* but not for *P. spumarius*. Sowing *Poaceae* proved to be consistently effective for *P. spumarius*, although yielded inconsistent results for *N. campestris* (i.e., was efficient during the first year but not upon the second year of sowing these species in the same experimental plot) [83].

### 4.3. Insecticide Use to Control the Vector

Besides interventions to remove the ground vegetation, different insecticides and natural compounds directly targeting the nymphs have also been tested [84]. Field trials conducted in Italy showed that among the products tested, neonicotinoids and pyrethroids consistently caused significant reductions of nymph populations. The other products tested (i.e., buprofenzin, sweet orange essential oil, kaolin, and zeolite) contributed to reductions of the number of nymphs (compared to the non-treated ground vegetation) but at lower effectiveness than the systemic insecticides. Additionally, the application of mineral oil (for eggs) and pelargonic acid tested in a single trial led to the lowest mortality rates for both spittlebug species [85].

Laboratory testing carried out in Spain [84] showed that pyrethroids (deltamethrin and λ-cyhalothrin) provided good control of *P. spumarius* nymphs and were fast acting (95% of mortality in 24 h). Sulfoxaflor (Closer^®^) exhibited similar efficacy at 48 and 72 h but it was slow acting. In contrast, pymetrozine and spirotetramat caused low mortality rates in nymphs. Higher efficacy was achieved when natural pyrethrins were combined with 1% or 3% of piperonyl butoxide: the mortality rate increased from approximately 30% (pyrethrin alone) to 95% (pyrethrin+ piperonyl butoxide) regardless of the amount of piperonyl butoxide added.

Overall, the testing of measures against the nymphal populations in Italy and Spain confirmed pyrethroids as the most efficacious class of insecticides, in line with previous data collected for the sharpshooter leafhopper *Homalodisca vitripennis* Germar (Hemiptera: Cicadellidae) [86]. Indeed, these studies disclosed important information on several alternative products, including synthetic and natural insecticides, which can be used in combination or as alternatives to mechanical interventions for integrated management of the spittlebug populations in areas where *X. fastidiosa*-infections occur or pose a serious threat (i.e., buffer zones, areas close due to outbreaks, etc.). Regarding the control of adults, different insecticides have also been tested on olives. The data available to date are mainly from trials carried out in Italy [87], where testing has been performed under semi-field conditions, i.e., by caging a pre-fixed number of adult spittlebugs on olive branches, with mortality rates assessed at different times after spraying.

Similarly to the results recorded for nymphs, synthetic pyrethroids (deltamethrin and lambda-cyhalothrin) and neonicotinoids (imidacloprid, acetamiprid, thiamethoxan) showed the highest efficacy against adults, causing increased mortality (with rates ranging from 76.7% to 100% at 3-DAT), with persistence up to 15–20 DAT (days after treatments) for pyrethroids and 20–25 DAT for neonicotinoids. Organophosphorus insecticides (chlorpyrifos-ethyl and chlorpyrifos-methyl) yielded lower mortality rates or inconsistent results, except for the applications based on phosmet, which caused mortality rates ranging from 69% to 82.5% and persistence up to 15 DAT [87].

Promising results were also obtained for cyantraniliprole (anthranilic diamide) [88]. The mortality and persistence rates were in some cases higher than those recorded for the reference products (pyrethroids and neonicotinoids), whereas spinosad, abamectin, and sweet orange essential oil showed prompt effects but poor or no persistence. Nevertheless, no mortality was recorded after use of spirotetramat, flonicamid, pymetrozine, buprofenzin, natural pyrethrin, or azadirachtin [83].

Izquierdo and Sabaté [89] also evaluated different synthetic insecticides (pyrethroids, flupyradifuron, spirotetramat) against juveniles and adults of *P. spumarius*. Among the pyrethroids, delthamethrin showed a high knockdown effect against both developmental stages, although with limited persistence, while flupyradifuron showed a slower prompt effect but higher persistence [89]. As with the data collected in Italy, the authors did not register insect mortality upon applications based on spirotetramat.

In almost all experimental trials against the adults on olives, the tested formulations were sprayed onto the canopy; however, information on the effectiveness of the insecticides using other modes of application is very limited. Dongiovanni et al. [88] reported comparative field trials where dimethoate and imidacloprid were supplied by spray applications and trunk injections. The results showed (i) a slower effect of the formulations injected into the trunk than the effect (mortality) recorded for the same formulation applied by spraying and (ii) inconsistent data in terms of persistence; nevertheless, no significant increase in the persistence of the active ingredients was recorded upon trunk injections, as would be expected.

It is worth noting that neonicotinoids have been used in several experimental trials as “terms of reference”, with the aim of finding alternative formulations showing similar levels of efficacy, although their use (i.e., the three active ingredients clothianidin, imidacloprid, and thiamethoxam) has been recently banned or almost prohibited in the EU (Regulations 2013/485 and 2018/783) [90,91]; therefore, more safe products should be developed to reduce *P. spumarius* populations.

Appropriate timing of the applying insecticides is a crucial aspect for the effectiveness of the control strategies against the vector. The results of surveys carried out in infected Italian olive groves [92] confirmed that *X. fastidiosa*-positive spittlebugs are detected soon after the emergence of the adults in early May, with the incidence increasing throughout the season; therefore, for an effective and sustainable control strategy, efforts should be made in the early phase of the adult season in an attempt to reduce as much as possible the number of adults visiting the infected olive canopies. Moreover, Bodino et al. [82] recently showed that the transmission efficiency may also increase in autumn, suggesting that spittlebugs visiting olive canopies late in the season, even if seldom, could also contribute to the disease spread. Nevertheless, inoculation events taking place late in the season can contribute to spreading infections over higher distances [80]. Interestingly, the experimental results gathered by Bodino et al. [82] showed that *P. spumarius* is not a very efficient vector if compared to *H. vitripennis*, the most effective sharpshooter vector in North American pathosystems, although population level and preference for olive plants can compensate for low efficiency. As such, the authors concluded that it is even more crucial to greatly reduce populations of this spittlebug in olive groves (preferably at the nymphal stage), since the number of feeding insects is probably the most important factor determining successful transmission and the quick spread of the pathogen.

### 4.4. Natural Enemies

Although lacking knowledge of potential natural enemies hampers the effective implementation of biological control measures [93], surveys for natural enemies of spittlebugs have been intensified during the past few years in Europe. Reis et al. [94] reported the occurrence of egg parasitoids in Portugal. More recently, the occurrence of the egg parasitoid *Ooctonus vulgatus* Haliday was reported in Corsica [95]. The predation dynamics of the generalist predator *Zelus renardii* Kolenati on *P. spumarius* were reported by Liccardo et al. [96]. Furthermore, Molinatto et al. [97] reported the infestation of field-collected spittlebugs by the parasitoid fly *Verrallia aucta* Fallén. Data for these natural enemy–vector interactions are preliminary and additional studies are needed to fully understand the degree of parasitoid–prey specificity to avoid non-target effects associated with any augmentative biocontrol measure.

### 4.5. Insect Repellent

Although very limited, attempts to reduce the transmission events have also been performed in olive groves in Italy [88]. Specifically, the effectiveness of kaolin was evaluated in a new olive plantation in the *X. fastidiosa*-infected area to assess its impacts as a potential insect repellent. Plants were protected (on a calendar basis) for three years during the whole season when adults are present. Applications with imidacloprid were used as reference insecticide control with respect to untreated control plants. The detection of the first infections was delayed by 6 months in the kaolin-treated plants and by 2 years in the imidacloprid-treated plants. Although a slower progression of the infections was detected among the treatments, after 3 years from planting no differences were recorded between treated and untreated plants. Even so, the delayed infections positively correlated with symptom onset, i.e., first shoot dieback on the untreated plants appeared already during the first year, while it was recorded after two years in the kaolin-treated plants and after three years on the plants sprayed with imidacloprid [88]. Overall, this experiment showed that the use of kaolin and imidacloprid did not prevent infections of healthy plants exposed to infective spittlebugs, although amelioration of the impacts of the infections was observed in the short period, probably as a consequence of the delayed infections and lower numbers of transmission events on the treated plants. These results further highlight the difficulties in finding an effective control for adult spittlebugs, reinforcing the need to put in place measures for the control of the juvenile forms.

### 4.6. Pheromones and Volatiles as Control Measures

Within the framework of developing control measures to include in integrated pest management (IPM) strategies, some studies have investigated innovative approaches, i.e., exploring the manipulation of the feeding and sexual behavior of *P. spumarius*. Although pheromones are not common in spittlebugs and are unlikely to be used as monitoring and control tools, recent studies on the structure of antennal sensilla of the spittlebug allowed the identification of chemoreceptors [98] and provided evidence of the functionality of antennal sensilla in *P. spumarius* adults [99]. In this latter study, the authors demonstrated the capability of the male and female olfactory systems to selectively perceive a variety of volatile compounds with a possible info-chemical role that may modulate *P. spumarius* intra- and interspecific interactions; however, it should be pointed out that among the 50 compounds tested in this work, only four compounds elicited significantly different responses between males and females, suggesting a general similarity between sexes in terms of antennal sensitivity. In a similar study, the behavioral responses of *P. spumarius* to a selection of essential oils and aromatic plants were investigated. The results showed good correlations between the bioactivity of odor sources and the negative and positive host preferences, demonstrating the capability of the peripheral olfactory systems of both sexes to perceive volatile compounds [100]. Indeed, these bioassays clearly indicated that males and females of *P. spumarius* respond differently to the same volatile blend. Although the recorded behavioral effects need to be confirmed under field conditions, these results may allow for semiochemical-based control strategies (i.e., push-and-pull or attract-and-kill strategy) to be developed in the future and for the use of essential oils to be extended for sustainable control of the *X. fastidiosa* vector.

Regarding the manipulation of sexual behavior, interesting investigations are ongoing to characterize the emission of vibrational signals by males and females. The first detailed description of the vibrational signals with specific roles within the mating behavior of *P. spumarius* was reported by Avosani et al. [101]. The authors concluded that even if further research is needed to identify an efficient signal and the most suitable strategy for field application, these preliminary results open up the possibility to manipulate *P. spumarius* behavior through artificial playbacks and for the future development of low environmental impact control practices, for example by attracting males into a trap using a specific vibrational signal.

## 5. Plant Breeding as a Sustainable Solution

Olive species have large (more than 900 cultivars) genetic and phenotypic variability in the Mediterranean area [102]. As soon as the epidemic started spreading in Apulia, several field observations indicated that while the more widely represented cultivars Ogliarola salentina and Cellina di Nardò were highly susceptible to *X. fastidiosa*, traits of resistance were found in the cultivar Leccino. Indeed, in trees of this cultivar, a lower incidence of infections and limited canopy desiccations were observed.

Studies of the plant response to the infection [10,103] showed that in xylem tissues of field-grown Ogliarola salentina and Leccino olives: (i) the bacterium population size of Leccino is 100 times lower (4 × 10^4^ vs. 2 × 10^6^ CFU/mL) than that of Ogliarola salentina; (ii) Leccino resistance is reproducible in greenhouse with artificial *X. fastidiosa* infections, while both susceptible cultivars show severe symptoms; (iii) genes associated with plant cell wall remodelling are altered in both cultivars, while receptor-like kinases and proteins and drought-associated genes are upregulated in Leccino and Ogliarola salentina, respectively. Similar molecular interactions were observed in grapevine [104,105] and citrus [106,107] infected by *X. fastidiosa*, and importantly receptor-like kinases are present in the PDR1 locus (pathogen disease resistance locus 1), as identified in the *X. fastidiosa*-resistant *Vitis arizonica* Engelm. [108,109]. The PDR1 trait was introgressed in *V. vinifera* L., and recently five bred grapevine cultivars were released on the market, indicating breeding for resistance as a promising strategy for the management of *X. fastidiosa* infections [110].

Leccino resistance in the field was confirmed in additional studies reporting a differential ionomer composition with respect to Ogliarola salentina [41]; an increase in quinic acid (a lignin precursor) in the Leccino response to the infection [12]; a possible role of the xylem anatomy in the resistance [111,112], as also reported in grapevine [108] and citrus [113]; and the role of biofilm and plant tylose response [114,115]. Further resistance traits were found in the cultivar FS17^®^, characterized by low bacterial population size and limited desiccations [116]. Several other olive cultivars are currently under evaluation regarding susceptibility to infections in the field or artificial conditions within the framework of the XF-ACTORS Project [117,118]. Long-term studies of resistance stability and crop yield are lacking, while their envisaged epidemiological benefits were confirmed as being reduced acquisition and transmission of the bacterium by *P. spumarius* when the inoculum source was Leccino or FS17^®^ [119].

A collaboration between the CNR-IPSP and an agronomist or olive oil producer is ongoing to evaluate the adoption of the grafting of Leccino on the susceptible cultivars Ogliarola salentina and Cellina di Nardò to save centennial trees within the framework of Project ResiXO, funded by the Apulian regional authority. In the same project, olive seedlings naturally grown in the epidemic area and surviving the infection have been selected and are now under screening to confirm the observed resistance and to evaluate their technological (i.e., olive and olive oil productions) characteristics, again pursuing a strategy of breeding for resistance to cohabit with the bacterium (Figure 2, bottom).

### Integrated Pest Management (IPM)

Since “there are still no risk reduction options that can remove the bacterium from the plant in open field conditions “, the core of IPM strategies used to control *X. fastidiosa*, when it is established in an area, relies on “early detection and rapid application of phytosanitary measures, consisting among others of plant removal and vector control” [26] (Figure 2, bottom). This declaration, therefore, evokes the two main pillars of “containment” measures of *X. fastidiosa* in the Apulian outbreak, namely the legislative (phytosanitary) measures and their application by means of an IPM strategy.

The crucial points of the IPM approach are a strategy of vector control, efficient and prompt detection of the bacterium, the removal of infected plants in the buffer zone, and the search for and adoption of resistant or tolerant species and cultivars. Fundamental to the design and implementation of such an IPM approach has been the study of the *X. fastidiosa* pathosystem. In-depth studies carried out within the framework of EU-funded POnTE [120] and XF-ACTORS [121] projects, as well as several regional projects, have allowed new and continuous knowledge to be generated on the biology and epidemiology of the disease (i.e., host range of the local *X. fastidiosa* strain, ecology of the transmitting vector(s), plant species and cultivar susceptibility to the bacterium) and diagnostic tools for laboratory (qPCR, ELISA) and early detection using remote sensing technologies; however, more effort is still needed to provide a comprehensive and sustainable solution. Tests of different biocontrol agents, mineral, and chemicals to target *X. fastidiosa* or the insect vector are needed, along with more plant breeding.

## 6. Conclusions and Future Prospects

In this review, we have described the history of *X. fastidiosa* infection in Europe, mainly on olive trees, and the actions that so far have been taken to fight the outbreak. Several solutions have been developed to reduce *X. fastidiosa* infection and to combat its insect vectors, in addition to breeding new resistant plant lines and using several approaches related to IPM. Although significant studies having been carried out, the *X. fastidiosa* disease is still spreading in olive groves. Traditional olive farms may change to the cultivation of other plants, with deep and detrimental impacts on the landscape, society, and European cultural heritage. There is an urgent need to work on new strategies and solutions and to examine and adapt EU and government regulations. We need further initiatives to develop sustainable solutions that will be effective under different pedo-climatic conditions. The EU-BBI-JU project BIOVEXO [122], in which academic and RTO partners, as well as industry leaders, SMEs, and farmers work together, aims to develop sustainable solutions targeting *X. fastidiosa* and its insect vector. Other projects dealing with basic and applied research on *X. fastidiosa* are also needed. XF-ACTORS and POnTE have provided enormous amounts of scientific knowledge; the Life Resilience [123] and CURE-XF [124] projects are currently evaluating and developing solutions as well.

Joint efforts such as those exemplified by EU-wide initiatives and projects will certainly lead to a better understanding of the aetiology of the *X. fastidiosa* disease and will provide a solid basis for arriving at new, environmentally sustainable, and finally successful solutions. This is in line with the ambitions set out in the European Green Deal, which aims to create a healthier and more sustainable EU food system through a 50% reduction in the use of chemicals and more hazardous pesticides by 2030. Presently, olive trees in Southern Europe are still at risk, and the disease might continue to spread to further areas; hence, visionary thinking and novel strategies are of utmost importance in all approaches to plant pest and disease control. It could take years before effective solutions are found, and farmers may have hard times ahead due to the disease burden affecting their crops and livelihoods. Scientists, farmers, and industry leaders, as well as regional, national, and international authorities, must join efforts and maximize their endeavors to save these olive trees and ensure their continued survival.

## Figures and Tables

**Table 1:** Reported occurrence ^1^ of *Xylella fastidiosa* subspecies in olive (*Olea europaea* L.) trees in the EU.

Geographic Area	*Xylella fastidiosa* Subspecies	Sequence Type
Apulia, Italy	*pauca*	ST53
Provence-Alpes-Côte d’Azur, France	*pauca*	ST53
Provence-Alpes-Côte d’Azur, France	*multiplex*	Unknown
Ibiza, Balearic Islands, Spain	*pauca*	ST80
Mallorca, Balearic Islands, Spain	*multiplex*	ST81
Madrid Community, Spain	*multiplex*	ST6
Porto Metropolitan Area, Portugal	*multiplex*	ST7

^1^ Data are those reported in the latest release of the *Xylella* spp. host plant database [4] and updated to 31 December 2020.
